# Supplementary material for: The association between osteopontin and tuberculosis: A systematic review and meta-analysis
Source: PLoS One. 2020 Dec 2;15(12):e0242702. doi: 10.1371/journal.pone.0242702 (PMC7710079; doi:10.1371/journal.pone.0242702)
Supplement: S1 Table — (DOCX) [file pone.0242702.s002.docx]

**S1 Table. Meta-regression analysis for potential sources of heterogeneity.**

| **Variable** | ***P* value** |
| --- | --- |
| Country (high burden and others) | 0.084 |
| Sample size | 0.981 |
| Gender | 0.978 |
| Average age | 0.968 |
| Tuberculosis type (PTB and others) | 0.893 |
| Immune status (non-immunocompromised and others) | 0.181 |
