# Supplementary figures and images for: The association between osteopontin and tuberculosis: A systematic review and meta-analysis
Source: PLoS One. 2020 Dec 2;15(12):e0242702. doi: 10.1371/journal.pone.0242702 (PMC7710079; doi:10.1371/journal.pone.0242702)

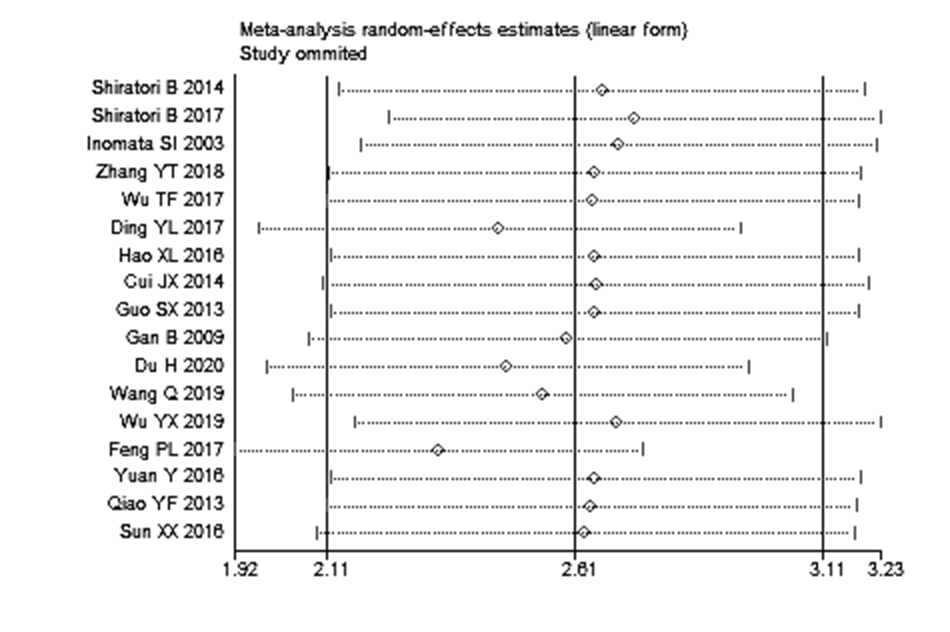

Supplement: S1 Fig — (TIF) [file pone.0242702.s003.tif]

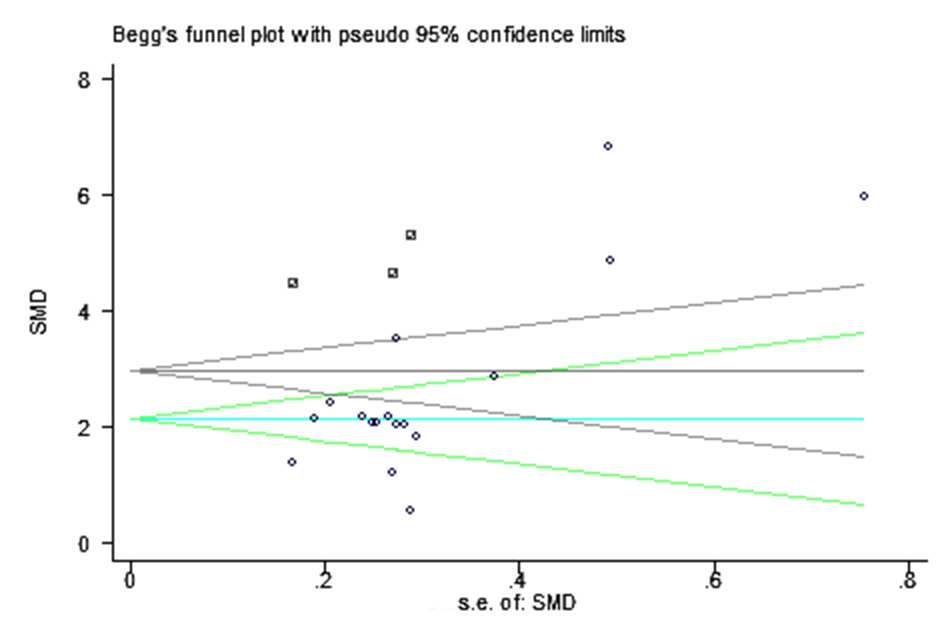

Supplement: S2 Fig — (TIF) [file pone.0242702.s004.tif]
